# 

**Published:** 1892-05-14

**Authors:** 


**Notices of Births, Deaths, and Marriages,** are inserted in this column at a charge of 2s. 6d. for an announcement not exceeding four lines.

### NOTICE TO CORRESPONDENTS.

All MS., Letters, Books for Review, and other matters intended for the Editor should be addressed The Editor, The Lodge, Porchester Square, London, W.

The Editor cannot undertake to return rejected M.S., even when accompanied by stamped directed envelope.

**Notice to Advertisers and Subscribers.**

All Advertisements, Orders for Copies of The Hospital, and Business Communications should be addressed to The Publisher, *not to the Editor*, The Hospital, 140, Strand, W.C.

The Cost of Subscription, post free, is as follows:—Per week, 2½d.; per quarter, 2s. 6d.; per half-year, 5s.; per annum, 8s. 6d. Foreign:—Per annum, 10s. 8d.; payable, in all cases, in advance.

**"Burdett's Hospital Annual," 1891—1892.**

Third year of publication. 600 pp. Boards, gilt lettering. A most complete guide to British and Colonial Hospitals, Dispensaries, Nursing and Convalescent Institutions, and Asylums. Price 3s. 6d. Published by The Scientific Press (Limited), 140, Strand, W.C.

**NOTICE.—The Index for Vol. X. (ending September, 1891), is on sale at the Office of this Journal, price 2½d., post free.**

### Scraps and Gleanings.

A fatal case of cholera is reported to have occurred at St. Denis, near Paris.

The twenty-fifth annual season of the Brompton Hospital entertainments ended with a most enjoyable concert.

Mr. John Cuckow, a resident of Lenham, Kent, has lately celebrated his hundredth birthday. Mr. Cuckow maintains his faculties practically unimpaired.

The Lord Mayor has received on behalf of the Hospital Sunday Fund a cheque for £105 in payment of legacy (duty free), by the executors of the late Mr. H. E. Price, of West Brighton.

The Irish Distressed Ladies' Fund has received from the executors of the late Mrs. Jane Anne Hibbert a donation of £50; and from the London Knot of the Friendly Brothers of St. Patrick £10 10s.

Patrick O'Sullivan, one of the men sentenced to penal servitude for life for complicity in the murder of Dr. Cronin, at Chicago, has died in the prison hospital at Joliet. He asserted his innocence to the last.

To commemorate the marriage of Lord Chelsea, his father, Earl Cadogan, has recently sent a cheque for £500 to the Chairman of the Vestry of the Parish of Chelsea, to be used for the benefit of the sick and poor.

Mr. Charles E. Flower, of Stratford-on-Avon, who gave £30,000 to the Shakespeare Memorial in his native town, died suddenly while attending a meeting of the Warwick County Council. He was about 70 years of age.

A new piano for the benefit of the patients in the Royal Westminster Ophthalmic Hospital has been purchased by subscription. It was first used at an entertainment given last week, when Lady Colin Campbell and other friends of the hospital lent their services.

Mr. Harry Furniss has returned to England completely restored to health. While in America he had an interview with the President, and inspected the Chicago Exhibition, and just before he left was royally entertained at a banquet given him by the Lotos Club.

A postman at Tattenhall, Cheshire, named Adams, has just completed a servitude of 21 years. During this period, with the exception of Sundays, he has walked on an average 20 miles a day. Adams estimates that his walking tour has extended to a little over 131,000 miles.

An excellent charity, which bears the title of the Diet Dispensary, has just been started on the other side of the Atlantic. The Dispensary provides, free of charge if necessary, or at a nominal charge when the applicant can afford to pay a little towards the cost, such nourishments as beef tea, new-laid eggs, and chicken broth.

The handsome new building, known as the John Horniman Convalescent Home for Children, Worthing, was opened on the 30th inst. The land and buildings have cost £10,000, and the Home is endowed with £20,000. The first instalment of patients—poor little waifs and strays from the East-end of London, were received a few days later.

An unpleasant experience has happened to Mrs. Double, of Colchester. As she was one day passing down the High Street she suddenly received a wound on the right side of the head from a small bullet, which penetrated to the bone. No report was heard, and it is conjectured that the shot may have come from an air gun. The lady is progressing favourably.

Lord Cadogan presided at the annual meeting of the Chelsea Hospital for Women. The report stated that the number of patients admitted into the hospital during the year was 494, while the attendances at the out-patient numbered 12,723. The annual subcriptions amounted to £1,956, and the donations to £782; while £600 had been received in bequests.

The Amateurs of Hayes, Middlesex, recently gave a performance of "Dream Faces" and "Larkin's Love Letter" in aid of the funds of the Cottage Hospital. The entertainment was a complete success from an artistic as well as a financial standpoint. In the interval between the pieces Mr. Alfred Copper gave a musical entertainment and also some thought-reading experiments.

Owing to an outbreak of scarlet-fever many of the children in the Orphanage of the Sisters of Charity, Carlisle Place, Westminster, have had to be sent to the Fever Hospital. Expensive measures have been ordered by the sanitary inspector, orders for needlework have perforce to be declined, and the Sisters are left without means to carry on the charitable work of their large establishment.

Baron Hirsch has decided to devote the whole of his turf winnings of last season, amounting in all to £7,000, to hospitals and similar institutions, without regard to religions and other distinctions. Amongst others, the North-West London Hospital, Kentish Town, is to receive £1,000, and University College, the London, the West London Hospitals, and the Hospital for Sick Children, Great Ormonde Street, will receive £700 each.

The total number of entirely *new* patients received at the Birmingham and Midland Ear and Throat Hospital in 1891 was 1,872. The total number of cases registered at the hospital during the year was 4,117. The Committee acknowledge with much gratitude the munificent bequest of the sum of £500 by Miss E. C. Villers Wilkes, of 10, Ampton Road, Edgbaston, who further enhanced the gift by bestowing it free of duty. Payment is expected this year.

August Wilhelm Hofmann, the celebrated professor of chemistry, has died at Berlin. After spending three years at Bonn, Professor Hofmann in 1848 was, on Liebig's recommendation, appointed Superintendent at the Royal College of Chemistry in London. In 1855 Professor Hofmann was appointed by the British Government a warden of the Royal Mint. In 1864 he was appointed to the chair of chemistry at Bonn, whence he was summoned to Berlin in the following year.

At the festival dinner of the British Home for Incurables, the Chairman said the Home received some forty patients; besides these, pensions were provided for 270 others, who were incurable but not wholly destitute. During the evening the Secretary announced donations amounting to £4,691. Of this amount Lord Egerton of Tatton contributed 100 guineas; Mr. F. Bevan, Chairman of the Board of Management, £500; and "Two Sisters," £1,000, for the endowment of a bed to be called, "Rest for the Weary."

### THE STUDENT OF MEDICINE.

[All communications for this Supplement should be addressed to The Editor of The Hospital, at 140, Strand, with the words, "Students' Column," written in the left hand top corner of the envelope.]

### APPOINTMENTS.

Arkle, C., M.D., M.R.C.P., appointed Pathologist and Curator of Museum at Charing Cross Hospital. Barratt, J. O. W., B.Sc., M.B.Lond., F.R.C.S., appointed Visiting Physician to the Birmingham Workhouse Infirmary. Bartlett, R.G., M.R.C.S., L.R.C.P.Lond., appointed House-Surgeon to the Teignmouth, Dawlish, and Newton Infirmary. Carter, A. H., M.D.Lond., F.R.C.P., M.R.C.S., appointed Visiting Physician to the Birmingham Workhouse Infirmary. Cole-Baker, G., B.A., M.B., B.Ch., B.A.O.Dub. Univ., L.M.Rot., appointed Assistant Master to the Coombe Lying-in Hospital, Dublin. Curtis, H. J., M.B., L.R.C.P.Lond., M.R.C.S.Eng., appointed Physician to University College Hospital. Dyson, W., B.A., M.D.Lond., appointed Honorary Consulting Physician to the Sheffield Hospital. Edwards, A. J., M.B., B.Ch.Vict., appointed House-Surgeon to the Manchester Southern Hospital and the Manchester Maternity Hospital. Gamgee, L. P., L.R.C.P.Lond., M.R.C.S., appointed Obstetric and Ophthalmic House-Surgeon to the Queen's Hospital, Birmingham. Gray, A. C. E., M.B., C.M.Edin., appointed House-Surgeon to the Royal Maternity and Simpson Memorial Hospital, Edinburgh. Ker, C. B., M.B., C.M.Edin., appointed House-Surgeon to the Royal Maternity and Simpson Memorial Hospital, Edinburgh. Lowe, G. M., M.D., C.M., L.R.C.P., L.R.C.S.Edin., appointed Honorary Surgeon to the Lincoln General Dispensary. McIlraith, C. H., M.A., M.B., C.M.Glas., appointed House-Surgeon to the Paddington Green Children's Hospital. Mathews, T. G., M.B., C.M.Edin., appointed House-Surgeon to the Cumberland Infirmary. Porter, Dr., S., appointed Honorary Consulting Physician to the Sheffield Hospital. Pritchett, S. J., L.R.C.P. Lond., M.R.C.S., appointed House Physician to the Queen's Hospital, Birmingham. Richards, H. M., M.B.Lond., M.R.C.S. Eng., L.R.C.P.Lond., appointed House-Physician to the University College Hospital. Short, T. S., M.B.Lond., M.R.C.S., appointed Visiting Physician to the Birmingham Workhouse Infirmary. Stead, G., L.R.C.P.Lond., M.R.C.S., appointed House-Surgeon to the Queen's Hospital, Birmingham. Templeman, C., M.D., B.Sc., appointed Surgeon to the Dundee Royal Infirmary. Underhill, C. E., M.B., F.R.C.P., F.R.C.S. Edin., appointed Physician to the Royal Maternity and Simpson Memorial Hospital, Edinburgh, vice D. Berry Hart, M.D.Edin. White, S., M.D., M.Ch.Irel., D.P.H.Camb., appointed Honorary Surgeon to the Sheffield Public Hospital and Dispensary. Williams, Mr. S., appointed House-Physician to the University College Hospital. Wilsden, A., M.B. and C.M.Aberd., appointed Junior Assistant Medical Officer to The Retreat, York. Wolverson, T., M.R.C.S., L.R.C.P., L.M., appointed Anæsthetist to the Wolverhampton and District Hospital for Women, Chapel Ash. Yearsley, P. M., M.R.C.S.E., L.R.C.P.L., appointed Senior House-Surgeon to the Western General Dispensary, Marylebone Road, N.W. Yuill, J. L., M.B., C.M.Glas., appointed Resident Medical Officer to the Monkwearmouth and Southwick Hospital, Sunderland.

### VACANCIES.

### Resident Medical Officers.

The following vacancies are announced this week, the amount of salary in each case being given after the name of the institution :

Addenbrooke's Hospital, Cambridge, Resident House-Surgeon—Salary £65 per annum, with board, &c.

Chelsea Hospital for Women, Fulham Road, S.W., Resident Medical Officer—Salary £80 per annum, with board, &c.

Derby County Asylum, Mickleover, near Derby, Assistant Medical Officer from June 18th to August 31st—Salary £2 2s. per week, with board, &c.

Hospital for Diseases of the Throat, Golden Square, W., Resident Medical Officer—Salary £50 per annum, with board, &c.

Hospital for Sick Children, Great Ormond Street, W.C., House-Physician, unmarried. Appointment for one year—Salary £60 per annum, with board, &c.

**Rotherham Hospital, Rotherham, Assistant House-Surgeon—Rooms, &c. Appointment for six months.**

Southport Infirmary, House-Surgeon, doubly qualified—Salary £100 per annum, with board, &c.

Western General Dispensary, Marylebone Road, N.W., Junior House-Surgeon, unmarried—Salary £50 per annum, with board, &c.

### Non-Resident Medical Officers.

Ballachulish Slate Works, Ballachulish, Medical Officer; unmarried—Salary £216 per annum, and general practice.

Hospital for Sick Children, Great Ormond Street, W.C., Assistant House-Surgeon. Appointment for one year—Salary £50 per annum.

Hospital for Sick Children, Great Ormond Street, Bloomsbury, W.C., Medical Registrar and Pathologist—Appointment for one year. Honorarium, 50 guineas at end of term.

Metropolitan Hospital, Kingsland Road, N.E., Vacancy on the Provident Department Staff. Honorarium at the rate of £100 per annum.

Northampton Friendly Societies' Medical Institute, Assistant Medical Officer; outdoor—Salary £400 per annum.

Sir Patrick Dun's Hospital, Dublin, Assistant Physician.

Sir Patrick Dun's Hospital, Dublin, Assistant Surgeon.

---

### PASS LISTS.

#### Royal Colleges of Physicians and Surgeons, Edinburgh, and Faculty of Physicians and Surgeons, Glasgow.

The quarterly examinations for the Triple Qualification in Edinburgh took place in April with the following results :—

First Examination.—Of 40 candidates who entered for the complete examination, the following 26 passed: T. A. W. Walker, Hyderabad; W. Shaw, Halifax, Yorks; W. G. McDowell, Liverpool; A. A. Bradburne, Liverpool; J. A. Kilvington, Yorkshire; H. G. Parker, Lincolnshire; W. J. S. Davis, London; I. Abd-el-Saiëd, Cairo; H. E. Birmingham, co. Galway; G. P. O'Connor, Cambridgeshire; J. J. Edgar, Northamptonshire; J. C. Ramsay, Peebles; G. T. H. De C. Lowe, Bangalore; F. A. Hadden, Rajahmundry; C. E. Page, Edinburgh; C B. Rossiter, Liverpool; J. Nelson, Limerick; R. Fairweather, Balfron; W. Pearson, Manchester; R. F. Yencken, Melbourne; J. C. Forbes, Edinburgh; J. D. Power, Cork; F. Jeeves, Belfast; A. Galloway, South Shields; D. Y. Clark, Abernethy, Perth; and J. B. Voortman, South Africa. Of eight candidates who entered for divisions five passed.

Second Examination.—Of 49 candidates who entered for the complete examination the following 24 passed :—C. S. Edwards, Staffordshire; W. G. McDowell, Liverpool; F. Isherwood, Lancashire; Holland May Harrison, Liverpool; G. Prentice, Carnwath; A. H. R. Porter, Victoria; Annie Florence Mary Cornall, Bristol; J. J. Harvey, Madras; J. Flynn, Queensland; T. Murphy, Cork; J. S. Maher, Tipperary; A. Crerar, Edinburgh; M. Hogan, co. Limerick; J. F. Colohan, Dublin; H. E. Birmingham, co. Galway; Grace Haxton Giffen, Morebattle; F. W. H. Wright, Warwickshire; J. A. Campbell, Victoria; E. P. Hasluck, Birmingham; C. Holding, Southampton; W. H. Griffith, Denbighshire; W. M. Morison, Stornoway; A. H. Brown, London; and P. Twomey, Ireland. Of 18 candidates who entered for divisions 13 passed.

Final Examination.—Of 101 candidates who entered for the complete examination the following 48 passed and were admitted L.R.C.P. and S.E. and L.F.P. and S.G.: G. Hepworth, Leeds; H. R. Preece, Cheltenham; C. F. G. Sixsmith, Cavan; A. G. Clark, Forfarshire; W. H. Knight, Lincolnshire; C. P. Felvus, Yorkshire; W. G McDowell, Liverpool; J. McKee, Greenock; R. Owen, Carnarvonshire; F. W. Mason, Leicester; R. R. R. Morrison, co. Cork; H. Ashton, Oldham; D. I. Jones, Carmarthenshire; W. Jameson, Carlow; J. F. Mitchell, Newtownards; R. H. F. Bostock, Leicester; E. W. F. Kirkman, Dublin; W. H. Walker, Ripon; F. A. Godfrey, Gibraltar; W. A. Collier, Dunkineeley; S. H. Heald, Wakefield; D. M. Steedman; Cape Town; S. J. Lightfoot, Limerick; C. A. Brough, London; I. W. Johnson, Nova Scotia; J. C. Loughridge, co. Antrim; T. Keays, co. Limerick; R. J. Blackham, Belfast; Elizabeth Adelaide Baker, South Africa; J. Kitchin, Elgin; H. G. Palmer, Portsmouth; D. C. Carnduff, Berhampore; R. E. Adamson, Cirencester; J. T. Spink, Leeds; E. S. Forde, Cork; M. Cahill, Cork; T. J. Lonergan, Melbourne; A. E. Hodges, co. Cork; J. R. M. Rae, Northumberland; F. T. Troughton, Kent; T. W. Norton, Brookhouse; E. Davies, Cardiganshire; C. R. Webster, York; J. R. Thomson, co. Antrim; B. S. Lockwood, Huddersfield; Susan Grace Dougall, Montreal; C. M. Coates, Bath; and A. J. A. Peters, Arbroath. Of 23 candidates who entered for divisions 15 passed.

#### Royal College of Physicians of London.

An ordinary comitia of the College was held on Thursday, April 28th, at five p.m. The minutes having been read and approved, the following gentlemen, who had passed the required examination, were then balloted for and admitted as Members: J. W. Campbell, M.B.Camb.; G. F. Crooke, M.B.Edin.; C. J. Macalister, M.B.Edin.; J. I. Parsons, M.D.Durh.; W. J. Sinclair, M.D.Aberd.; W. W. H. Tate, M.B.Lond. The licence of the College was granted to 132 gentlemen who had passed the required examinations. The following Members nominated by the Council were elected Fellows: D. W. C. Hood, M.D.Camb.; J. E. Ranking, M.D.Ox.; H. Sainsbury, M.D.Lond.; T. C. Fox, M.B.Lond.; W. A. Foxwell, M.B.Camb.; J. Phillips, M.B.Camb.; W. D. Halliburton, M.D.Lond.; L. E. Shaw, M.D.Lond.; W. Collier, M.D.Camb.; H. W. G. Mackenzie, M.B.Camb.; F. W. Mott, M.D.Lond.; and J. Reid, M.D.Aberd. The examiners for the Murchison Scholarship reported that none of the candidates were of sufficient merit to justify the award.

